# Ticket to Ride: A Longitudinal Journey to Health and Work-Attendance in the JD-R Model

**DOI:** 10.3390/ijerph18084327

**Published:** 2021-04-19

**Authors:** Benedicte Langseth-Eide, Joar Vittersø

**Affiliations:** Department of Psychology, UiT The Arctic University of Norway, 9019 Tromsø, Norway; joar.vitterso@uit.no

**Keywords:** work engagement, JD-R model, sick absence, employee well-being

## Abstract

The present study addresses one of the limitations of the JD-R model, namely, that analyses of the outcomes of the motivational process have largely focused on organizational outcomes and have neglected to investigate the associations between job resources, work engagement and health-related outcomes. Specifically, the aim of this paper is to show that health-related indicators may be outcomes of the motivational process in the job demands-resources (JD-R) model. We achieve this through a two-wave panel study with a two-year time lag. The results provide longitudinal evidence that two well-established job resources (i.e., social support and feedback) predicted work engagement, that work engagement was negatively related to sick leave and that this relation was mediated by subjective health. By showing that health-related indicators could also be outcomes of the motivational process in the JD-R model, we have strengthened the model.

## 1. Introduction

The identification and implementation of factors that can contribute to improving and enhancing employee health and well-being is needed in order to create and provide healthy workplaces [1]. However, although flourishing organizations and engaged employees in general promote work attendance and positive employee health, these processes are not fully understood.

In the well-established job demands-resources (JD-R) model [2], health-related indicators have most often been measured as outcomes of the health-impairment process, whereas organizational outcomes have been linked to the motivational process. It has been argued that one of the limitations of the JD-R model is the lack of investigation of the relations among job resources, work engagement and health-related outcomes [3]. Recent research has shown that the JD-R model could, in addition to organizational outcomes, include health-related outcomes in its motivational process [3]. Even though it has been suggested to expand the motivational process in the JD-R model with regard to health-related outcomes, it is necessary to perform studies that can validate this expansion.

### 1.1. The Motivational Process in the JD-R Model

The JD-R model assumes that job demands and job resources (i.e., working conditions) will negatively or positively impact organizational outcomes and employee well-being. This happens through two distinct psychological processes: either the health-impairment process or the motivational process [2]. The health impairment process posits that job demands lead to burnout [4] or workaholism [5,6], which in turn leads to ill health. On the other hand, the motivational process posits that job resources first lead to work engagement and thereafter to positive organizational outcomes [7,8].

#### 1.1.1. Job Resources Leads to Work Engagement

Job resources are those physical, psychological, social or organizational aspects of a job that may stimulate personal growth, development and learning; that may assist in achieving work goals; and that may reduce job demands and the associated psychological and physiological costs [9]. Job resources may be intrinsically motivating by facilitating growth, development and learning [2] and thus function to satisfy basic needs, such as the needs for autonomy, relatedness and competence [10,11]. For example, social support may fulfill the need to belong [12], and suitable feedback may foster learning, which increases job competence [8]. The same job resources may also be extrinsically motivating by providing tools or concrete information that contribute to goal attainment. Further, social support may function as hands-on assistance to handle momentary work overload (i.e., reduce job demands) to reach work goals [13], while feedback can provide concrete information that may contribute to goal achievement. Hence, both social support and feedback enhances the possibility that an employee successfully will achieve his or her goals at work [9]. In addition, being in a resourceful work environment may stimulate a desire to dedicate one’s capabilities and effort to the job task [4], which increases the likelihood that the tasks will be completed and that work goals will be achieved. Whether these resources (i.e., social support and feedback) satisfy basic needs or contribute to achieving work goals, the outcome is positive, and it is likely that work engagement will emerge [9,14]. Work engagement is defined as a “positive, fulfilling work-related state of mind that is characterized by vigor (that is, high levels of energy and mental resilience while working), dedication (referring to a sense of significance, enthusiasm and challenge), and absorption (being focused and happily engrossed in one’s work)” [15] (p. 46).

Empirical studies have consistently shown a positive relationship between several job resources, such as job control, autonomy, skill variety and learning opportunities and work engagement [8,16,17,18]. Additionally, Schaufeli and Bakker [9] used structural equation modeling (SEM) to reveal that three job resources, namely, performance feedback, social support and supervisory coaching, predicted work engagement. The relationship between job resources and work engagement is compatible with the job characteristic theory [19]. The job characteristic theory emphasizes that certain core job characteristics (i.e., skill variety, task identity, task significance, autonomy and feedback) will lead to different positive work-related outcomes, of which intrinsic motivation corresponds with work engagement. Also, self-determination theory [20] suggests that job resources satisfy the basic human needs for autonomy, competence and relatedness. The fulfillment of these needs leads to increased intrinsic motivation and optimal functioning, which is essential for psychological health and well-being. Conservation of resources (COR) theory [21,22] is also compatible with the notion that job resources are associated with work engagement. COR theory suggests that resources evolve in cycles, meaning that various types of resources are likely to accumulate over time because the existence of resources may bring additional resources [23]. As stated in the COR theory, people strive to obtain, retain and protect their resources, including job resources. Individuals with strong resource pools invest resources for future gains and thus experience a gain cycle. On the other hand, those who do not have access to strong resource pools have an increased likelihood of experiencing resource loss (i.e., loss cycle). Hence, gaining resources increases the likelihood that additional resources will be acquired, which in turn increases work engagement. Empirical evidence for an upward spiral of resources and work engagement has been presented. For example, Dicke et al. [24] showed that resources had positive long-term effects on work engagement two years later among German teachers. Simultaneously, work engagement led to higher levels of resources over time. In a similar vein, Reis, Hoppe and Schröder [25] found a reciprocal relationship between work engagement and mental health among psychotherapists. In addition, they found that work engagement was both a predictor and outcome for autonomy, learning opportunities and task variety (i.e., job resources). Furthermore, Schaufeli and Salanova’s [15] study with managers revealed that engagement predicted higher levels of job resources, such as social support, autonomy, learning and performance feedback the following year. Together, these findings suggest that engaged employees are capable of mobilizing their own personal resources and job resources, which in turn nourish forthcoming engagement, and onward.

#### 1.1.2. Work Engagement Leads to Better Self-Reported Health and Reduced Sick Leave

In well-being research, there is a growing interest in the associations between positive work-related conditions and states, and health outcomes. Work is identified as an important health determinant [26], and gainful employment is considered to foster good health. For example, Keyes [27] showed that flourishing employees had a lower risk of cardiovascular disease and reported fewer days of sick leave than their less-flourishing colleagues.

Convincing empirical support has been provided for the motivational process of the JD-R model, which moves from job resources through engagement to positive organizational outcomes [7,14,28]. It has been shown, for example, that work engagement is positively associated with positive work-related attitudes, commitment to the job and organization, and better performance at work. However, the link between job resources and health-related outcomes, such as subjective health and sick absence, via work engagement has rarely been investigated [3]. However, there is some empirical evidence of a positive association between job resources and work engagement and health-related outcomes. Hakanen and Schaufeli [29] showed that engagement was positively related to life satisfaction. Airila et al. [3] revealed that engagement mediated the relationship between job and personal resources and work ability, in which the latter includes being healthy enough to perform the job. Moreover, Langseth-Eide [5] revealed that workaholism was negatively related to work-related health but that work engagement was positively related to work-related health, although both workaholics and engaged employees worked overtime hours. Additionally, previous studies have revealed that engaged employees report fewer psychosomatic complaints [30]; suffer less from head pain, cardiovascular problems and abdominal pain [9]; and report better self-reported health [31].

This is in line with COR theory [21,22] that describes pathways from job resources via work engagement to health in long term gains. People employ their resources not only to deal with stress, but also to have a pool of resources for future needs. These resources are salient in creating well-being (i.e., work engagement) and in enhancing health. In the long term, individuals that have access to greater resources will experience future resource gains, and this will contribute to protect against stress and as a consequence they will be better protected against illness and ill-being. To summarize, COR theory presumes that increased levels of resources will be beneficial for well-being and health in the long term.

Healthy employees are less absent from work, and there is empirical evidence that health mediates the relationship between job-related states (such as work engagement) and sick leave. For example, Schalk [32] showed in a longitudinal study that workplace attitudes (i.e., job satisfaction and organizational commitment) were negatively related to sick leave and that this relationship was mediated by employee health. Further, based on their summarization of previous studies, Pousette and Hanse [33] proposed that the relationship between particular work attitudes and sick leave was mediated by health. Thus, there is some empirical evidence to suggest that engagement can predict reduced sick absence and that this relationship is mediated by subjective perceptions of health.

#### 1.1.3. Aim of the Study

With the present study we aim to show that the motivational process in the JD-R model, which starts with job resources that give rise to work engagement, may, in turn, lead to positive health-related outcomes. Although the proposed processes in the JD-R model have been replicated numerous times, a great majority of these studies have been performed with cross-sectional data. Our contribution includes both concurrent and longitudinal (panel) data.

Accordingly, we hypothesize:

**Hypothesis** **1a:** *Employees’ job resources (i.e., social support and feedback) predict their level of work engagement*.

**Hypothesis** **1b:** *Employees’ level of work engagement in a given point in time (T1), directly affects their level in job resources (i.e., social support and feedback) in a subsequent time point (T2)*.

**Hypothesis** **2a:** *Work engagement is negatively related to sick leave*.

**Hypothesis** **2b:** *The relationship between work engagement and sick leave is mediated by employees’ self-reported health levels*.

## 2. Materials and Methods

### 2.1. Sample and Procedure

The data for the present study were collected as part of a work environment survey among public employees from many workplaces and a wide variety of professions in a municipality in Norway (e.g., teachers at elementary schools and art schools, lawyers, cleaners, public health nurses, nurses, physiotherapists, librarians, bureaucrats, social workers, engineers, firemen, librarians, IT advisors, translators, janitors and administrative personnel). The broad variety of professions and workplaces is favorable regarding external validity. Participants were invited by e-mail with a link to an electronic questionnaire at both T1 and T2 two years later. A total of 1544 and 1503 employees participated in the survey at T1 and T2, respectively. The participants could make their T1 and T2 information identifiable on a voluntary basis. A total of 185 participants completed both questionnaires and made themselves identifiable and could therefore be included in the longitudinal analyses; 27% (*N* = 50) of the participants were men, and 73% (*N* = 135) were women. The mean age at T1 was 33.4 (*SD* = 10.05).

### 2.2. Measures

#### 2.2.1. Feedback

To measure feedback, a five-item scale developed by Kuvaas [34] was employed. An example item is “I receive frequent and continuous feedback on how I do my job”. The Cronbach’s alpha coefficients were 0.87 and 0.85 at T1 and T2, respectively. The responses were provided on a 7-point Likert scale (1 = Strongly disagree, 7 = Strongly agree).

#### 2.2.2. Colleague Support

Colleague support was measured using a four-item subscale from the Survey of Perceived Organizational Support [35]. An example item is “My colleagues really care about my well-being”. The Cronbach’s alphas were 0.94 and 0.89 at T1 and T2, respectively. The responses were provided on a Likert scale ranging from 1 (=Strongly disagree) to 7 (=Strongly agree).

#### 2.2.3. Work Engagement

Work engagement was measured with the nine-item version of the Utrecht Work Engagement Scale [36]. The items cover three aspects of work engagement: vigor, dedication, and absorption. Sample items are “At my work, I feel bursting with energy” (vigor), “I am enthusiastic about my job” (dedication) and “I am immersed in my work” (absorption). The response alternatives ranged from 0 (Never) to 6 (Every day). Exploratory factor analysis with maximum likelihood conducted with the data from the present study did not indicate a clear three-dimensional model, and neither did following confirmatory maximum likelihood factor analysis. For this reason, a one-dimensional mean score variable based on the nine items was computed and used in the subsequent analyses. The Cronbach’s alphas were 0.94 at T1 and 0.95 at T2.

#### 2.2.4. Self-Reported Health

A single item was used to measure the participants’ subjective health: “How would you describe your present health?” [37]. The response alternatives were Very poor, Poor, Average, Good or Very good. This single-item measure of self-reported health has previously been used in numerous studies [38,39]. Self-reported health has been closely related to somatic and psychological complaints in several previous studies and has also proven to be a predictor of objective health measures and mortality [40,41]. It has been argued that this single-item measure of subjective health is correlated strongly with other direct or indirect measures of health and has good test-retest reliability, demonstrating a high degree of construct validity [42].

#### 2.2.5. Sick Absence

To assess the participants’ sick absence, we asked participants how many times (spells/episodes, not days) they had been absent from work due to sickness during the past 12 months. Sick absence can be assessed as spell-, person-, or time-based measures. Sick absence spells, often referred to as sick leave episodes, are common events in the general population. Sick absence spells have a skewed distribution, in which short-term spells are common, whilst long-term spells take place to a smaller extent. In previous reviews of measurements of sickness absence, Hensing [43] and Hensing et al. [44] suggested the following five measures for sick absence: frequency, length, cumulative incidence, incidence rate and duration. Frequency was suggested as a basic measure. They argue that it is suitable to apply frequency as a measurement when studying workplaces as it can provide an overview of the burden of sickness absence within a limited study population.

### 2.3. Analyses

We computed the internal consistencies, descriptive statistics, and intercorrelations of the study variables using the PASW 25.0 program (IBM, Armonk, NY, USA).

To test our hypotheses, we conducted SEM analyses using the Mplus 8.0 software package (Muthén & Muthen, Los Angeles, CA, USA) [45]. The fit of the models were assessed with the chi-square test, root mean square of approximation (RMSEA), comparative fit index (CFI), Tucker-Lewis index (TLI) and standardized root mean square residual (SRMR). It is suggested that RMSEA values below 0.07, SRMR values below 0.08, and CFI and TLI values greater than 0.95 indicate good fit [46].

## 3. Results

The descriptive statistics, Pearson’s correlations and Chronbach’s alpha of the study variables are presented in Table 1. All variables were normally distributed within the limits of a skewness less than |2| [47]. None of the mean scores differed significantly across time points (all *p* values from paired-sample *t*-tests > 0.270). As expected, the cross-sectional correlations between the two job resource variables and the work engagement variable were moderately high, ranging between *r* = 0.24 and *r* = 0.43 (*p*’s < 0.01). The associations between these variables and the sick leave variables were negative and much smaller, in the range of *r* = −0.06 to *r* = −0.16, all *p*’s > 0.05, except for the association between feedback and sick leave at T1, (i.e., *r* = −0.16) which was significant at *p* = 0.037. Self-reported health and sick leave correlated negatively and significantly, *r* = −0.20, *p* = 0.008, and *r* = −0.33, *p* < 0.001, at T1 and T2, respectively. Self-reported health at T1 also correlated negatively with sick leave at T2, *r* = −0.27, *p* = 0.001.

**Figure 1:**
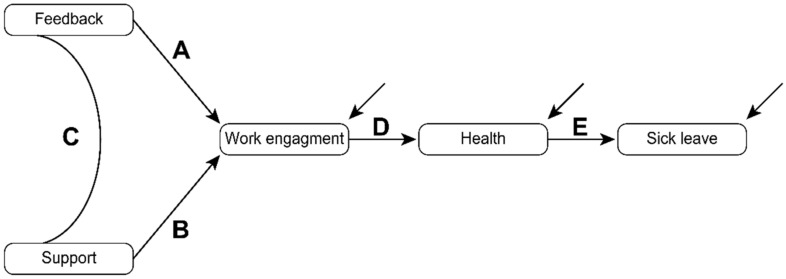
Cross-sectional path model.

The variables were fitted to the path model depicted in Figure 1. All models received acceptable goodness of fit (Table 2). The standardized regression coefficients (betas) were all significantly different from zero (*p* < 0.01) and were in the range from β = −0.14 (*p* < 0.001) for path E (cf. Figure 1 and Table 3) in the complete sample at T1 to β = 0.41 (*p* < 0.001) for path A in the longitudinal subsample at T1.

To determine whether the associations in the full samples at T1 (*N* = 1544) differed from those in the subsample of participants who completed the questionnaires at both time points (*N* = 185), we constrained all coefficients in the full sample T1 model to be equal to those in a model with the longitudinal data (i.e., the *N* = 185 sample at T1). We repeated the procedure for two models at T2 (i.e., compared results based on the full *N* = 1503 sample, with data from the *N* = 185 sample).

Using a multigroup strategy, we first inspected the differences in chi-squares for the two T1 model models, and next for the two T2 models. The chi-square difference at T1 was not significant, Δχ^2^(5) = 3.09, *p* = 0.686. Similarly, the chi-square difference at T2 was not significant either, Δχ^2^(5) = 3.03, *p* = 0.695.

Hence, with regard to the *associations* between the present study variables, we assumed that they were the same for those of the participants who completed both questionnaires as compared to those who only completed either the T1 or the T2 questionnaire.

Regarding the size of the regression weights, the results from the models were consistent with the results from the zero-order correlations. No direct effect from job resources to health and sick leave were included in the model, and we did not observe any direct effect from work engagement to sick leave.

**Figure 2:**
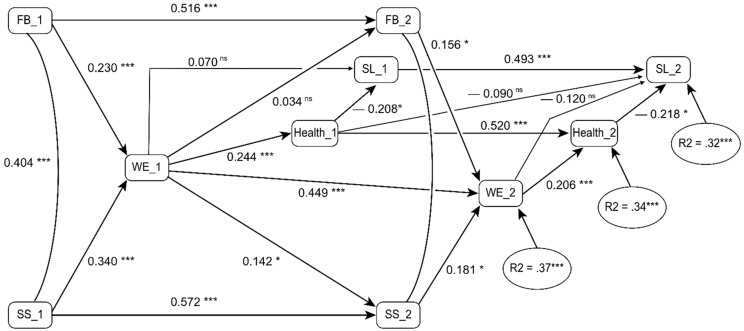
Longitudinal model. FB = Feedback; SS = Social Support; WE = Work engagement; SL = Sick leave episodes; 1 = T1; 2 = T2, * = *p* < 0.05; *** *p* < 0.001; ns = non-significant.

Figure 2 shows our final model, which integrated information from both data waves. The model adequately fit the data with χ^2^(25), *N* = 184) = 34.92, *p* = 0.089, CFI = 0.98, RMSEA = 0.05 [0.00−0.08], SRMR = 0.05. The model showed the cross-sectional stability between variables and crossover effects within variables. For example, the cross-sectional paths from health to sick leave were β = −0.21 and β =−0.22 at T1 and T2, respectively (both *ps* < 0.01). A direct path from health at T1 to sick leave at T2 was nonsignificant, β = −0.09, *p* = 0.259.

Our first hypothesis (H1a) stated that job resources predict work engagement, which is confirmed in the path from feedback to work engagement (β = 0.23, *p* = 0.001) and from social support to work engagement (β = 0.34, *p* < 0.001) in Figure 2. Our second hypothesis (H1b) was partly supported. It suggests that work engagement at T1 predicts job resources at T2, which it does for social support (β = 0.14, *p* = 0.021) but not for feedback (β = 0.03, *p* = 0.617).

Our final hypotheses are that work engagement is negatively related to sick leave (H2a), and that this relationship is mediated by health (H2b). Hypothesis 2a was not supported, since no significant paths between work engagement and sick leave were found (*ps* > 0.073). Hypothesis 2b was supported, however, since the indirect effects at both T1 (β = −0.07, *p* = 0.044) and T2 (β = −0.06, *p* = 0.034) were significant and in the expected direction. Longitudinally, a small but significant indirect effect was found from work engagement at T1 through general health at T1 on sick leave at T2 (β = −0.02, *p* = 0.047).

## 4. Discussion

The aim of this paper was to show that the JD-R model could be expanded by including health-related indicators as outcomes in the motivational process. Hence, we examined antecedents (i.e., job resources) and consequences (i.e., health and sick leave) of work engagement within the framework of the JD-R model.

As expected, we found longitudinal evidence that social support and feedback predicted work engagement (H1a). Our findings support the main notion of the motivational process of the JD-R model, namely, that job resources have a positive effect on employee well-being [2]. Hence, it is likely that a resourceful job environment enhances the chances of having engaged workers. These findings are in line with the assumption in the COR theory of an accumulation process resulting in resource gains. When employees hold resources they value, they are more likely to continue to invest resources, which in turn increases work engagement. Our final model also supports previous studies that have revealed a positive association between job resources and work engagement [31,48].

Drawing on the reciprocal process described in COR theory, we also hypothesized that work engagement at T1 would predict job resources at T2 (H1b). This hypothesis was only partially confirmed. We found a significant relationship between work engagement at T1 and social support at T2, but a nonsignificant relationship between work engagement and feedback at T1 and T2. There might be several reasons for this finding. One issue to consider is the relatively high stability of work engagement [49]. Due to the relatively stable nature of many psychological constructs, the predictors will fail to account for any additional variance in the outcome variable. Time lags that are too long may also lead to the underestimation of the true causal impact [50]. The two-year follow-up period may have been suitable to investigate the association between work engagement and social support among colleagues. Often, employees are colleagues for several years, and social support, which also has a relational aspect, may therefore not be very vulnerable to longer time lags between measurement points. On the other hand, it is possible that the two-year time lag is unsuitable to investigate the association between feedback and work engagement. Feedback is a transaction between the leader and the employee and is often tied to job tasks and performance [34]. It may be that levels of feedback change more during a two-year period than the social relations among colleagues, and feedback may therefore be more vulnerable to long time lags between measurements. Future studies should investigate the longitudinal relationship between feedback and work engagement in more detail. However, the overall results were meaningful and support the motivational process in the JD-R model.

Finally, we wanted to investigate the inclusion of health-related indicators as outcomes of the motivational process in the JD-R model. The results did not provide longitudinal evidence that work engagement directly led to reduced sick leave (H2a), but a significant mediating effect was found via self-reported health (H2b). Thus, it seems that engaged workers experience better subjective health than less engaged workers and that they are less absent from work. Hence, our findings support the pathway described in the COR theory in which job resources are positively related to health-related outcomes via engagement through long-term gains.

There might be several reasons why engaged workers report better health and are less absent from work. Previous studies have revealed that compared to less-engaged workers, engaged workers recover from their workdays better [51] and more often experience positive emotions [52]. Engaged workers also report that they more often participate in leisure-time activities that help them relax and detach from work, such as sports and exercise, social activities, and hobbies [53]. Additionally, Schaufeli and Bakker [10] found that engaged workers suffered less from self-reported headaches, stomach aches, and cardiovascular problems. Hence, work engagement may lead to something else beyond positive organizational outcomes. Our results provide evidence that the JD-R model could also be used to more broadly predict positive health, as well as the (negative) health outcomes that often follow the health-impairment pathway.

### Limitations and Future Research

The current study has some limitations that should be mentioned. We used questionnaires to collect the data, and there are some limitations to this method. First, the results are based entirely on single-source self-ratings, which may imply that the relationships among the variables are due to common method variance. However, applying a longitudinal design has been shown to reduce the problem with unmeasured third variables and common method variance [54]. We also conducted Harman’s single-factor test, and the results showed that common method variance did not pose a problem in this data set [55]. Still, future studies could enhance the explanatory power of the model by including other measures. For example, observer ratings have previously been used successfully to study working conditions [31] and could be applied. Future studies could also attempt to apply a mixed-methods design, in which a representative sample of the participants that have answered the questionnaire are also interviewed. This may deepen and expand the findings. We also want to acknowledge that, although the study design was longitudinal, there was no experimental manipulation of independent variables and therefore we cannot make any causal inferences with confidence [56].

There are also limitations inherent in the measurement of subjective health. In questions about self-reported health, there is often a norm or benchmark attached to it. Participants may, for example, compare themselves to similar others (e.g., how my health is compared to others at my age) or take time into account (e.g., my current health status compared with one year ago). In order to overcome these challenges, objective measures could be applied. In addition, our study may display a selection bias called “the healthy worker effect”, that is, only the strongest and healthiest employees stay in the work force while those who are unhealthy leave working life. However, empirical studies suggest that problems with nonresponse are more severe for estimations of population means than for estimations of associations [57].

There are also limitations in the measurement of sick leave. The reliability of the measure of sick leave in this study may have been reduced due to memory bias, since what we actually measured was employees’ recalled sick leave. Again, objective measures, such as absence registers, may be employed in future studies. Another important factor is that the sickness absence of employees in Norway is completely financially compensated during their first year. Hence, employees on sick leave experience no loss of income. It cannot be ruled out that other results would be obtained in countries where sickness absenteeism leads to income loss. In these countries, it might be that disengaged employees are less inclined to be absent from work. This restricts generalizations of the findings beyond Norwegian employees. Future studies should attempt to replicate the study in other countries that have different financial policies regarding compensation during sick leave in order to overcome this problem.

Finally, there are some limitations regarding the sample in our study. Although the participants represent a large variety of professions and workplaces within the municipality, they are all from the same geographical area and have the same overall employer. Thus, there might be that our findings cannot be generalized to other communities. Future studies should attempt to investigate the associations between job resources, work engagement, health and sick leave in both the private and public sector, in different occupations and workplaces and in different areas of the world.

## 5. Conclusions

In summary, our results provide firm longitudinal evidence that job resources promote work engagement and that engaged workers experience good health and are less absent from work. Therefore, our results support the expansion of the motivational process of the JD-R model to include not only organizational outcomes but also health-related outcomes. Although the responsibility for own health is individual, it is also a societal responsibility to create conditions that enable people to influence their health and well-being. The notion that work engagement is a predictor of positive subjective health that, in turn, leads to reduced sick leave emphasizes the importance and implications of facilitating a resourceful work environment.

## Figures and Tables

**Table 1:** Descriptive statistics, Pearson’s product-moment correlations and Cronbach’s alphas (diagonally presented) for feedback, social support work engagement, self-reported health, and sick absence spells.

	Range	*M*	*SD*	Sk	T1(1)	T1(2)	T1(3)	T1(4)	T1(5)	T2(1)	T2(2)	T2(3)	T2(4)	T2(5)
Time 1														
T1(1). Feedback	1 to 7	3.57	1.46	0.22	(0.87)									
T1(2). Social support	1 to 7	5.68	1.31	−1.28	0.41 ***	(0.94)								
T1(3). Work engagment	1 to 7	5.67	1.22	−1.26	0.37 ***	0.43 ***	(0.94)							
T1(4). Self-reported health	1 to 5	3.94	0.76	−0.35	0.06	0.13	0.24 **	N.A.						
T1(5). Sick absence spells	1 to 13	3.05	1.83	1.83	−0.16 *	−0.06	−0.12	−0.20 **						
**Time 2**														
T2(1). Feedback	1 to 7	3.63	1.39	1.39	0.53 ***	0.24 **	0.22 **	0.03	−0.16 *	(0.85)				
T2(2). Social support	1 to 7	5.62	1.22	1.22	0.31 ***	0.64 ***	0.39 ***	0.14	−0.16 *	0.30 ***	(0.89)			
T2(3). Work engagement	1 to 7	5.57	1.25	1.25	0.24 **	0.40 ***	0.55 ***	0.19 **	−0.18 *	0.31 ***	0.40 ***	(0.95)		
T2(4). Self-reported health	1 to 5	3.88	0.79	0.79	0.05	0.13	0.15 *	0.56 ***	−0.22 **	0.05	0.09	0.31 ***	N.A.	
T2(5). Sick absence spells	1 to 13	3.08	2.08	2.08	−0.02	−0.08	−0.08	−0.27 ***	−0.54 ***	−0.14	−0.04	−0.26 ***	−0.33 ***	N.A.

Note. *M* = Mean, *SD* = Standard Deviation, Sk = Skewness, * *p* < 0.05, ** *p* < 0.01, *** *p* < 0.001 (two-tailed tests).

**Table 2:** Goodness-of-fit measures for the model depicted in Figure 1, fitted to the full samples at T1 and T2 and to the longitudinal samples at T1 and T2.

Model	χ^2^(5)	*N*	*p*	CFI	RMSEA (90% CI)	SRMR
Model 1 (T1)	15.04	1544	0.010	0.96	0.04 [0.02–0.06]	0.03
Model 2 (T1)	4.67	185	0.457	1.00	0.00 [0.00−0.10]	0.04
Model 3 (T2)	18.11	1501	0.003	0.97	0.04 [0.02−0.06]	0.02
Model 4 (T2)	8.88	185	0.114	0.95	0.07 [0.00−0.13]	0.05

χ^2^(df) = Chi-square (degrees of freedom), CFI = Comparative Fit Index, RMSEA = Root Mean Square of Approximation, SRMR = Standardized Root Mean Square Residual.

**Table 3:** Standardized regression coefficients (β’s) and 95% confidence intervals (CIs).

Path	T1			T1 Longitudinal			T2			T2 Longitudinal		
	β	LL-CI	UL-CI	β	LL-CI	UL-CI	β	LL-CI	UL-CI	β	LL-CI	UL-CI
a	0.37	0.30	0.43	0.34	0.21	0.47	0.28	0.23	0.33	0.34	0.21	0.47
b	0.22	0.15	0.28	0.23	0.10	0.37	0.27	0.22	0.31	0.21	0.08	0.34
c	0.30	0.23	0.35	0.41	0.28	0.53	0.30	0.26	0.34	0.30	0.12	0.43
d	0.29	0.23	0.25	0.24	0.11	0.38	0.25	0.20	0.29	0.31	0.13	0.44
e	−0.14	−0.20	−0.10	−0.20	−0.35	−0.06	−0.19	−0.25	−0.14	−0.33	−0.51	−0.20

## Data Availability

The data are protected thus not openly available.
